# No Evidence That Cognitive and Physical Activities Are Related to Changes in EEG Markers of Cognition in Older Adults at Risk of Dementia

**DOI:** 10.3389/fnagi.2021.610839

**Published:** 2021-03-19

**Authors:** Daria Laptinskaya, Olivia Caroline Küster, Patrick Fissler, Franka Thurm, Christine A. F. Von Arnim, Iris-Tatjana Kolassa

**Affiliations:** ^1^Clinical and Biological Psychology, Institute of Psychology and Education, Ulm University, Ulm, Germany; ^2^Department of Psychology, University of Konstanz, Konstanz, Germany; ^3^Department of Neurology, Ulm University, Ulm, Germany; ^4^Clinic for Neurogeriatrics and Neurological Rehabilitation, University- and Rehabilitation Hospital Ulm, Ulm, Germany; ^5^Psychiatric Services of Thurgovia, Academic Teaching Hospital of Paracelsus Medical University Salzburg, Muensterlingen, Switzerland; ^6^Faculty of Psychology, TU Dresden, Dresden, Germany; ^7^Division of Geriatrics, University Medical Center Göttingen, Göttingen, Germany

**Keywords:** coherence, lifestyle, dementia, mismatch negativity, cognitive training, physical training, electrophysiology

## Abstract

An active lifestyle as well as cognitive and physical training (PT) may benefit cognition by increasing cognitive reserve, but the underlying neurobiological mechanisms of this reserve capacity are not well understood. To investigate these mechanisms of cognitive reserve, we focused on electrophysiological correlates of cognitive performance, namely on an event-related measure of auditory memory and on a measure of global coherence. Both measures have shown to be sensitive markers for cognition and might therefore be suitable to investigate potential training- and lifestyle-related changes. Here, we report on the results of an electrophysiological sub-study that correspond to previously published behavioral findings. Altogether, 65 older adults with subjective or objective cognitive impairment and aged 60–88 years were assigned to a 10-week cognitive (*n* = 19) or a 10-week PT (*n* = 21) or to a passive control group (*n* = 25). In addition, self-reported lifestyle was assessed at baseline. We did not find an effect of both training groups on electroencephalography (EEG) measures of auditory memory decay or global coherence (*p*s ≥ 0.29) and a more active lifestyle was not associated with improved global coherence (*p* = 0.38). Results suggest that a 10-week unimodal cognitive or PT and an active lifestyle in older adults at risk for dementia are not strongly related to improvements in electrophysiological correlates of cognition.

## Introduction

The number of people with cognitive deficits and dementia is growing due to increasing life expectancy and demographic change. Thus, the meaning of the detection of pathological cognitive decline as well as its prevention and slowing down became an increasingly important issue. For decades the assessment of cognitive decline in healthy aging and in dementia had been mainly based on neuropsychological data. However, recently surrogate biomarkers in cerebrospinal fluid and neuroimaging have evolved. In our recent studies we described two sensitive electroencephalography (EEG) markers for cognition. These are resting-state global coherence (Laptinskaya et al., 2019) and a novel mismatch negativity (MMN) index for auditory memory decay, namely ΔMMN (Laptinskaya et al., 2018).

Resting-state global coherence cognition was defined as the coherence over the whole skull at rest in the frequency range between 4 and 30 Hz. Coherence represents functional coupling between brain regions or single electrode pairs. A wide range of studies reported alterations in resting-state EEG coherence in dementia (e.g., Knott et al., 2000; Stevens et al., 2001; Adler et al., 2003). The most prominent change is attenuated coherence in the alpha and beta frequency range in fronto-parietal and the temporo-parietal coupling (see Babiloni et al., 2016 for a review). Recent studies suggest that these changes might already occur in mild cognitive impairment (MCI; Michels et al., 2017). In line with previous study results investigating frequency- or region-specific coherence, we found global coherence to be a sensitive EEG marker for global cognition (Laptinskaya et al., 2018).

The MMN is one of the most widely investigated ERP components and is elicited when a presentation that has been automatically predicted by the central nervous system is violated (Näätänen et al., 1978, 2011). In an auditory paradigm the MMN can be evoked when in a sequence of equal tones a deviant tone is presented. The MMN represents two, closely linked, processes: auditory discrimination ability and auditory memory. With increasing interstimulus interval (ISI, the distance between the standard and the deviant tone) MMN provides more information on the auditory memory trace (for a review see Bartha-Doering et al., 2015). Many previous studies have shown attenuated MMN amplitude, especially after long ISIs in dementia (Pekkonen et al., 1994; Schroeder et al., 1995; Papadaniil et al., 2016) as well as in MCI as a possible prodromal stage of dementia (Lindín et al., 2013; Ji et al., 2015; Papadaniil et al., 2016). However, no MMN marker for cognition has been established yet. This might be due to the fact that the comparability between study results is difficult because of the methodological difference, such as different deviant types and ISI lengths. To address this challenge, we built a difference score between MMNs after short and after long ISIs. The difference score (hereafter referred to as ΔMMN) takes individual differences in auditory discrimination ability as well as auditory memory into account and has been shown to reflect cognition or even the cognitive decline over a period of 5 years (Laptinskaya et al., 2018).

Beneath new diagnostic tools for cognition, intervention methods which are capable to prevent or slow down cognitive decline are of great clinical and scientific interest. Previous studies suggest that cognitive and physical activity may benefit cognition or even delay dementia (Livingston et al., 2020). Regarding the impact of physical and cognitive activity on cognition, specific training programs and an active lifestyle can be distinguished. The last one is often defined as the number of regularly performed activities. While some studies reported positive training effects on cognition (e.g., Hess et al., 2014; Lampit et al., 2014; Edwards et al., 2016; Groot et al., 2016), others failed to find beneficial effects (e.g., Williamson et al., 2009; Barnes et al., 2013). Training factors such as the combination of cognitive and physical training (PT) aspects (e.g., Fissler et al., 2013; see Gheysen et al., 2018 for a review and a recent meta-analysis), longer training periods, and variability in training tasks (cf., Fissler et al., 2013) seem to be important determinants for training success. Results regarding active lifestyle are more consistent than results on training interventions: the majority of studies reported a positive association between an active lifestyle and cognition. But the comparability between training and lifestyle studies is limited. Training effects on cognition often refer to time limited experimental designs. In contrast, lifestyle cognition studies are associative in nature and consider longer time periods. Furthermore, lifestyle cannot be experimentally manipulated. To fill this scientific gap, we systematically investigated the effects of specific training programs with lifestyle cognition associations for 10 weeks in older adults with increased risk of developing dementia (cf., Küster et al., 2016). We found that persons with a more active lifestyle showed benefits in cognitive performance post-training when compared to persons with a less active lifestyle. In contrast, neither a 10-week auditory cognitive training (CT) nor a 10-week PT showed beneficial effects on cognition. We concluded that the lifestyle might be beneficial for cognition more than the short-term training programs by higher variability in cognitive demands, enjoyment, fun, and intrinsic motivation.

Until now the biological mechanisms of positive effects of cognitive and physical activity on cognition are not well understood. Understanding the mechanisms of treatment is decisive to develop individualized treatments that specifically target brain changes that underlie cognitive symptoms. In the present study, we examine the association between lifestyle and changes in EEG markers for cognition as well as the effect of a 10-week auditory CT and a 10-week PT on the two EEG parameters. The results might give further insights into electrophysiological mechanisms which underlie the positive link between cognitive and physical activity and changes in cognition. In the long term, this knowledge could be used for individualized therapies that are based on biomarker profiles of patients with cognitive impairment. The aim of the present study was to investigate the association between lifestyle and EEG markers for cognition. Furthermore, we tested the hypothesis, whether specific training programs are suitable to enhance global cognition and ΔMMN.

## Materials and Methods

### Participants

The study was approved by the ethics committees of both study centers, the University of Konstanz and Ulm University, Germany. The study was part of a controlled clinical trial investigating the effect of physical exercise and auditory CT on cognition as well as on electrophysiological and biological parameters (Küster et al., 2016, 2017; Fissler et al., 2017; Laptinskaya et al., 2018, 2019). Prior to the study, all participants provided written informed consent. Subjects were recruited in the Memory Clinic of Ulm University Hospital, Germany and the Center for Psychiatry Reichenau, Germany, or via public advertisements. The detailed description of the inclusion and exclusion criteria can be found in Küster et al. (2016). In brief, inclusion criteria were: 55 years of age or older and subjective or objective memory impairment, fluency in German language, stable anti-dementia and antidepressant medication, and independent living. Exclusion criteria were as follows: probable moderate or severe dementia [Mini Mental State Examination; MMSE (Folstein et al., 1975) < 20] and history of other neurologic or psychiatric disorders (except mild to moderate depression). Furthermore, participants with severe hearing or visual impairment, and physical impairment which could restrain the participation in the training programs were also excluded.

**FIGURE 1:**
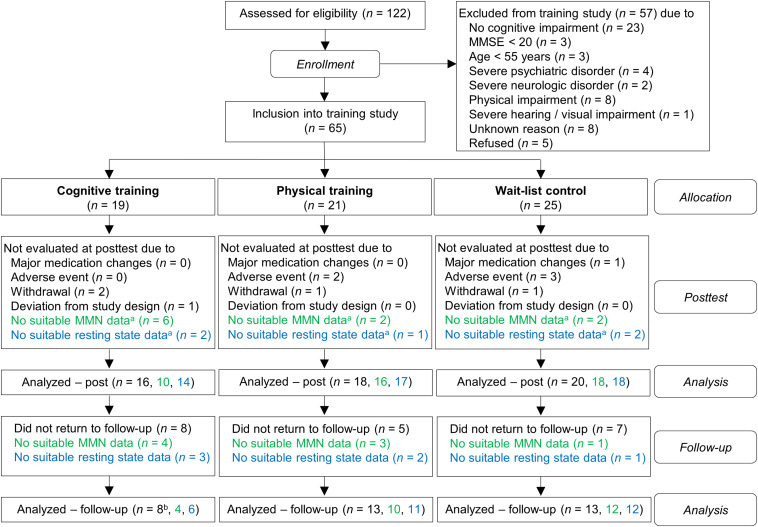
Flow chart of participants within study groups. Information written in green refers to ΔMMN data and information written in blue refers to global coherence data. ^a^Pre-training, post-training, or both. ^b^For one subject, the global coherence data for all three time-points (pre, post, follow-up), but no cognitive data, were available.

Out of 122 participants who were screened for eligibility, due to exclusion and drop-outs 65 subjects were included in the study (for the detailed flow of participants see Figure 1). Subsequently, the participants were assigned into one of the three groups: auditory CT (*n* = 19), PT (*n* = 21), and wait-list control (WLC; *n* = 25). In order to minimize differences in age, gender, education, and cognitive status (MMSE) between groups, we used a minimization approach. The investigators were blind to the subjects’ group allocation. In rare cases this was not maintained because the participants disclosed their group assignment during neuropsychological assessment. Notably, because of drop-outs, the number of evaluated training data sets varied for MMN and for coherence analysis (for drop-out reasons see Figure 1).

According to classification criteria (see Supplementary Presentation 1) the participants were classified as having subjective memory impairment (SMI, *n* = 12), amnestic cognitive impairment (aMCI, *n* = 23), non-amnestic cognitive impairment (naMCI, *n* = 14), or probable dementia (AD_*p*_, *n* = 5).

### Procedure

Four weeks prior to the commencement of the intervention and within a slot of one or two appointments, details of sociodemographic, cognitive and lifestyle data, as well as electrophysiological recordings were collected. Prior to the beginning of EEG recordings, individual hearing thresholds were assessed using in-house software PyTuneSounds (Hartmann, 2009). The training or the waiting period started 1–4 weeks after the pre-training test and lasted 10 weeks. Post-training tests were carried out 1–4 weeks after the end of the intervention. Potential long-term effects were examined in follow-up tests 3 months after the post assessment.

### Neuropsychological Assessment

All participants completed the following assessments: the Alzheimer’s Disease Assessment Scale–cognitive subscale (ADAS; Ihl and Weyer, 1993), phonemic and semantic word fluency as well as TMT part A and B of the Consortium to Establish a Registry for Alzheimer’s Disease–plus test battery, the subtests digit span and digit-symbol coding of the Wechsler Adult Intelligence Scale (Tewes, 1991). Furthermore, an adapted German version of the California Verbal Learning Test [German: Münchner Verbaler Gedächtnistest [MVGT, Munich Vebal Memory Test); Ilmberger, 1988] was conducted. Additionally, everyday cognition in an ecologically valid task was assessed using the working-memory subtest of the Everyday Cognition Battery (ECB computation span, Allaire and Marsiske, 1999). Verbal Knowledge Test (German: Wortschatztest) was used to assess crystallized abilities.

To reduce multiple testing and thus α-inflation and in order to assess latent cognitive function scores, a principal component analysis using the oblique rotation technique was performed across all participants. Using the Kaiser criterion (Eigen values ≥ 1.0) two components were extracted. One component showed high loadings of episodic memory scores, namely MVGT encoding, MVGT long-delay recall, and ADAS-free recall. The second component showed high loadings of attention and executive function scores, i.e., TMT part A and B, digit span forward and backward, digit-symbol, phonemic and semantic fluency, and ECB computation span. All variables were *z*-standardized and two component scores were built, one representing memory functions and the one representing attention and executive functions. The components represent the weighted average of those *z*-standardized variables with loadings of at least *a*_*ij*_ = 0.40 on the respective component. Additionally, a global cognition component score was calculated as the average of the two component scores. A more detailed description of the principal component analysis can be found in Küster et al. (2016).

### EEG Assessment

#### EEG Recording and Data Processing

The EEG was recorded using a high-density 256-channel HydroGel Geodesic Sensor Net (HCGSN; Electrical Geodesics, Inc.; Eugene, OR, United States). During data acquisition Cz served as reference and the data were sampled with 1,000 Hz. Resting state EEG as well as MMN EEG were collected in the same session after neuropsychological assessment, starting with the resting state EEG recording. During EEG recordings participants were seated comfortably in an electrically shielded room. To avoid drowsiness artifacts during resting state EEG, participants were instructed to keep their eyes open and to fixate on a chosen mark approximately at eye level on the opposite wall. Furthermore, to avoid blink and muscle artifacts, the participants were instructed to relax and to reduce blinking during both EEG recordings. There was a 5 min break between the recordings. During MMN EEG recording participants watched silent Charlie Chaplin videos while auditory stimuli were presented binaurally through stereo headphones at 50 dB above the individual hearing threshold. Since the MMN is elicited automatically without participants’ attention and it is preferable to keep attention effects low during MMN recording, participants were instructed to watch the video carefully and not to pay any attention to the sounds.

After recording the EEG, the data were imported into MATLAB (version 2015b; The MathWorks, 2015); the FieldTrip toolbox (version 20151012) was used for preprocessing.

#### Global EEG Coherence Analyses

Data were bandpass filtered in the range of 1-30 Hz (24 dB/octave), noisy channels were rejected, and the data were re-referenced to the average of all channels marked as good. For a better comparison between assessments, the noisy channels were compared between recordings and the same channels were used for coherence analyses for the pre-post (*M* = 220.65, SD = 20.76, range: 170–246) and the pre-follow-up comparison (*M* = 220.93, SD = 20.88, range: 170–246), respectively. Artifact-contaminated epochs were manually rejected. To avoid trial bias, 20 trials were randomly selected from clean epochs for each subject and assessment. Spectral coherence analyses were calculated as an index for functional connectivity assuming a linear coupling in brain activity. Spectral coherence analyses were applied using the *bsmart* implementation for MATLAB (Cui et al., 2008). As an index for global coherence, we calculated the spectral coherence for all pairs of electrodes and averaged all values for the 1–30 Hz frequency range as an index for global coherence.

#### ΔMMN Analyses

##### Procedure and paradigms

We used two different passive MMN paradigms. The paradigm order was counterbalanced between subjects. The Optimum–1 paradigm (Näätänen et al., 2004) focuses on auditory discrimination ability. Stimuli were presented with a constant SOA of 0.5 s. The Optimum–1 paradigm is suitable to detect MMN after five deviant types: duration, frequency, intensity, location, and a gap deviant. The paradigm presents 1,845 stimuli in three 5 min blocks. Every second tone was a standard tone. Thus, the probability for the standard tone was 50% and the probability for each deviant type was 10%. For the formation of a standard tone as such, a sequence of 15 standard tones was presented at the beginning at each block.

The Memory Trace paradigm (in accordance to Grau et al., 1998) uses long 3 s ISI and was developed to investigate the auditory memory trace. A total number of 462 stimuli are presented within three blocks of 6 min each. The same standard tone, duration and frequency deviant as in the Optimum–1 paradigm were used in the Memory Trace paradigm. As in the Optimum–1 paradigm 15 standard tones were presented at the beginning of each block for the standard tone formation. One to three standard tones were presented between two deviant tones. The number of standard stimuli and the ISI between standard tone (0.5, 1.5, or 3 s) were assigned pseudo randomly. The ISI between standard stimuli and deviant tone was constantly 3 s. Standard stimuli were presented with 66.2% probability and each deviant tone with a probability of 16.9%.

##### Detailed description of the stimuli

The standard tone was a harmonic tone of three sinusoidal partials of 500, 1,000, and 1,500 Hz with the second partial being 3 dB and the third being 6 dB lower in intensity then the first partial. The standard tone was 75 ms in duration including 5 ms rise and fall times. The duration deviant was 50 ms shorter in comparison to the standard tone. The gap deviant comprised a 7 ms silent gap (including 1 ms fall and rise times) in the middle of the tone. One half of all frequency deviants were 10% higher (partials: 550, 1,100, and 1,650 Hz) and the other half 10% lower in frequency than the standard tone (partials: 450, 900, and 1,350 Hz). Intensity deviants were 10 dB louder or lower than the standard tone (50% each). The location deviants had an interaural time difference of 800 μs to the left or to the right ear (50% each).

##### MMN analysis

For both paradigms the EEG data were bandpass filtered in the range of 1–35 Hz (24 dB/octave) and noisy channels were interpolated using the average method. Artifact-contaminated epochs were manually rejected. Finally, the data were re-referenced to the linked mastoids. As the largest MMN is often assessed at fronto-central EEG electrodes, and the averaging of electrodes with similar activity has been demonstrated to show more reliable results than the measure of single separate electrodes (Huffmeijer et al., 2014), the average voltage at FCz, Fz, and Cz was computed as mean MMN amplitude for all further analyses.

The number of averaged trials can influence the signal-to-noise ratio. Thus, to avoid these confounding effects, main analyses were repeated by building the ΔMMN from 50 randomly selected artifact-free trials for each assessment and each subject.

##### Calculation of ΔMMN

The difference score ΔMMN was defined by subtracting MMN after long ISI from MMN after short ISI, with higher values indicating less automatic auditory memory decay (cf., Laptinskaya et al., 2018). Thus, for the calculation of ΔMMN the MMN in both Optimum–1 and Memory Trace paradigms needed to be available. Since no MMN was observed after the frequency deviant in the Memory Trace paradigm in a previous study (Laptinskaya et al., 2018), we restricted the ΔMMN analyses to the duration deviant. The dataset of one person was excluded from analyses, as no Optimum–1 data were available.

### Assessment of Lifestyle

For the assessment of lifestyle we used the Community Healthy Activities Model Program for Seniors (CHAMPS) Physical Activity Questionnaire for Older Adults (Stewart et al., 2001). The questionnaire describes 40 daily life activities, each assigned to a cognitively challenging (e.g., solving crossword puzzles, reading), physical (e.g., swimming, running), or social domain (e.g., meeting friends and family, calls with friends and family). Each activity was rated by three of the authors (PF, OCK, DL) on a five-point rating scale from 1 (*no demands*) to 5 (*high demands*). Activities with a rating score above three points were categorized to the respective domain (see Supplementary Table 1). Cronbach’s α for authors’ ratings was very good: α_*Cronbach*_ = 0.92 for physical domain, α_*Cronbach*_ = 0.86 for cognitive domain, and α_*Cronbach*_ = 0.95 for social domain. The categorization was validated by ratings of 39 independent healthy older adults (MMSE ≥ 26, aged 64–90). The comparison between authors’ and seniors’ ratings revealed very high correlations for all domains: *r* = 0.87 for physical domain, *r* = 0.89 for cognitive domain, and *r* = 0.84 for social domain. If the chosen categories differed between authors and seniors the classification was adapted to the seniors’ opinion. Finally, the cognitive domain comprised 14 activities, the physical domain 17 activities, and the social domain 13 activities. Twelve activities could not be assigned to any domain because of an overall loading under three points.

Study participants were asked which typical activities they perform within a 4-week span. For each domain the number of performed activities was divided by the number of potential activities in this domain. Subsequently, the domain values were averaged and the final average value was regarded as the index for lifestyle activity.

### Training Interventions

#### Auditory Cognitive Training

As CT we employed the German adapted version of the Brain Fitness software provided by the Posit Science Corporation (San Francisco, CA; for more detailed training tasks description see Küster et al., 2016). It consists of six tasks which target working memory and auditory processing. Each exercise lasted approximately 15 min. One training session included four out of six training tasks and took 1 h in duration. The training difficulty was automatically adapted according to the participant’s task performance. Participants were asked to perform the 1-h session once a day, 5 days per week for a period of 10 weeks. Thus, the participants completed 50 training hours in total.

#### Physical Training

The PT was carried out in groups consisting of 5-10 participants and involved aerobic, strength, flexibility, coordination, and balance elements. The training was based on a training program that has shown positive effects in nursing home residents (Thurm et al., 2011). Each group met twice a week for a 1-h session over a period of 10 weeks. The difficulty of the training was adapted by two instructors. Furthermore, the participants completed three fixed homework sessions per week of about 20 min each with the same exercise elements as the main training. Homework sessions were documented and regularly checked by the instructors. Thus, in total the participants completed 20 training hours in the group setting and 10 training hours at home.

#### Wait-List Control Group

Participants in the WLC were asked to continue their daily routine as usual. After study participation, we offered them the participation in one of the training programs.

### Statistical Analyses

#### General Procedure and Descriptive Statistics

All statistical analyses were carried out with *R* (version 3.2.3; R Core Team, 2016) in *RStudio* (RStudio Team, 2015). In group comparisons, all model residuals were normally distributed according to the Shapiro-Wilk normality test; therefore, parametric tests were applied. Baseline group differences in continuous variables (demographics and outcome measures) were evaluated with univariate analysis of variance (ANOVA) models with intervention group (CT, PT, and WCT) as between group factor. Differences in gender distribution were analyzed with Pearson’s Chi-square (χ^2^)-test. Normal distribution of all models’ residuals was confirmed using the Shapiro-Wilk test (*W* statistic) and visual inspection (Q–Q plots). The statistical significance level (α) was set to 0.05 for all analyses.

#### Cognitive Progression of the Sample

These analyses were performed in accordance to Küster et al. (2016, p. 6, 8) and are repeated here to show the cognitive progression for the sample. The overall cognitive change was demonstrated for the cognitive component scores as well as for single test values. For this purpose, comparisons between post- and pre-training were performed. To adjust for possible retest effects, we calculated *z*-values based on baseline assessment for pre- and for post-training, respectively. Subsequently, the standardized post-value was subtracted from the pre-value and 95% confidence intervals were calculated. Finally, Group (CT vs. WLC and PT vs. WLC) × Time (pre vs. post) as well as Lifestyle (continuous) × Time interactions were performed for each cognitive outcome to test for possible differences in cognitive progression in dependence on group allocation or lifestyle. Training effects on cognitive progression were indicated by a significant Group × Time interaction, while positive associations between lifestyle and cognitive change were indicated by a significant Lifestyle × Time interaction.

#### Lifestyle Global Coherence Association and Training Effects on Global Coherence

We evaluated the association between active lifestyle and change in global coherence as well as training effects on global coherence by employing linear mixed-effects models using the nlme package 3.1.119 (Pinheiro et al., 2011). The model included Group (CT vs. WLC and PT vs. WLC) × Time (pre vs. post) as well as Lifestyle (continuous) × Time interactions as fixed effects in the same model, and Subject as random intercept with Global coherence as dependent variable. Significant Group × Time interactions reflected a training effect on global coherence, and significant Lifestyle × Time interactions demonstrated an association between lifestyle and global coherence. Hedges’ *g* was calculated by comparing the difference scores in the auditory cognitive or PT group and the control group, respectively. As secondary analyses, *t*-tests were carried out for pre-post comparison in global cognition for each group separately.

Nowadays, the most prominent coherence chance has been reported for the fronto-temporal and fronto-parietal area. Thus, as supplemental analyses, the same linear mixed-effects model was used to assess training-induced effects and lifestyle-associated changes in fronto-temporal and fronto-parietal coherence.

#### Training Effect on ΔMMN

As for global coherence, to explore training effects on ΔMMN, a linear mixed-effects model was modeled. It was restricted to Group (CT vs. WLC and PT vs. WLC) × Time (pre vs. post) as fixed effect with ΔMMN as dependent variable because we focused on the effect of auditory CT on ΔMMN and did not expect a positive effect of PT or a positive association between lifestyle and ΔMMN Significant lifestyle associations for the period of 10 weeks were revealed by a significant Lifestyle × Time interaction, while training effects on EEG indices were indicated by a significant Group × Time interaction. Hedges’ *g* was calculated to show effect sizes for the training programs. Therefore, the *z*-standardized difference score between pre- and post-training was built for the two interventions as well as for the control group. Hedges’ *g* was calculated by comparing the difference scores in the auditory cognitive or PT group and the control group, respectively. Positive effects indicate beneficial effects of the intervention.

The main analysis was repeated with ΔMMN calculated from constant 50 trials (for rare exceptions see Supplementary Presentation 1) for each subject.

Again, as secondary analyses, *t*-tests for paired samples were performed for pre-post comparisons in ΔMMN within each group.

#### Follow-up-Assessment

In a final step, all linear mixed-effects models were repeated, including the follow-up analysis as a third time point in the model (pre vs. post and pre vs. follow-up) to account for possible long-lasting training effects.

## Results

### Mismatch Negativity Analysis

The number of trials was left for averaging in the Optimum–1 paradigm as well as in the Memory Trace paradigm can be found in the Supplementary Material.

### Group Comparisons at Baseline

At baseline, the three study groups (CT, PT, and WLC) did not differ in demographic variables, cognitive data, lifestyle data, and in EEG parameter (see Table 1).

**TABLE 1:** Baseline characteristics and group comparisons for and between study groups.

	Cognitive training (*n* = 16)	Physical training (*n* = 18)	Wait-list control (*n* = 20)	*F* statistic	*p*
**Demographic data**					
Age (y.)	70.2 ± 5.8	73.7 ± 6.2	70.3 ± 5.5	*F*_(2,51)_ = 2.11	0.13
Gender (f./m.)	8/8	12/6	10/10	χ^2^_(2)_ = 1.35	0.51
Education (y.)	13.3 ± 4.0	14.2 ± 3.0	15.2 ± 3.7	*F*_(2,51)_ = 1.18	0.32
**Cognitive data**					
MMSE	27.8 ± 2.6	27.8 ± 1.7	28.2 ± 2.2	*F*_(2,51)_ = 0.14	0.87
SMI/naMCI/aMCI/AD_*p*_	3/4/8/1	6/4/7/1	3/6/8/3	χ^2^_(2)_ = 3.22	0.78
Global cognition (cs.)	0.08 ± 0.64	0.04 ± 0.62	−0.10 ± 0.82	*F*_(1,52)_ = 0.61	0.44
Memory (cs.)	−0.02 ± 0.83	0.16 ± 0.67	−0.11 ± 0.98	*F*_(1,52)_ = 0.15	0.70
Attention/executive functions (cs.)	0.19 ± 0.64	−0.08 ± 0.75	−0.08 ± 0.78	*F*_(1,52)_ = 1.14	0.29
**Lifestyle data**					
Number of reported activities	8.4 ± 3.4	8.7 ± 2.5	9.3 ± 2.5	*F*_(2,49)_ = 0.39	0.68
Variety of activities	0.27 ± 0.13	0.28 ± 0.09	0.30 ± 0.09	*F*_(2,49)_ = 0.60	0.55
**EEG data**					
Global coherence	0.36 ± 0.12	0.29 ± 0.18	0.29 ± 0.10	*F*_(2,46)_ = 1.20	0.31
ΔMMN	−1.26 ± 1.05	−1.46 ± 0.92	−1.33 ± 1.69	*F*_(2,41)_ = 0.09	0.92

*Values are means (M) ± standard deviations (SD).*

*SMI, subjective memory impairment; naMCI, non-amnestic mild cognitive impairment; aMCI, amnestic mild cognitive impairment; AD_*p*_, probable dementia; y., years; f., female; m., male; cs., component score. ΔMMN, the difference score between MMN for long and for short ISI for duration deviant. The data refer to complete sample without the exclusion of ΔMMN and global coherence missing data (see Figure 1). Data analyses accounting for the missing data did not change the results.*

### Cognitive Progression of the Sample

As already shown by Küster et al. (2016, p. 8), no positive training effects on cognition were found neither for the auditory cognitive nor for the PT program, *p*s ≥ 0.08 (see Table 2). In turn, participants who reported a more active lifestyle had a significantly better progression in global cognition and in memory functions over a period of 10 weeks in comparison to their less active counterparts, *p*s < 0.01 (see Table 2).

**TABLE 2:** Cognitive changes and group effects on cognition as well as lifestyle cognition associations over time (cf. Küster et al., 2016, p. 8).

	Difference post-pre [95% CI]			Group × Time		Lifestyle × Time	
			
	Cognitive training (*n* = 16)	Physical training (*n* = 18)	Wait-list control (*n* = 20)	*F* statistic	*p*	*F* statistic	*p*
**Cognitive measure**							
Global cognition (cs.)	0.20 [0.03, 0.37]	0.16 [0.01,0.30]	0.32 [0.22, 0.43]	*F*(2,48) = 2.64	0.08	*F*(1,48) = 18.77	<0.01
Memory (cs.)	0.34 [0.11, 0.57]	0.15 [−0.10, 0.40]	0.38 [0.19, 0.58]	*F*(2,48) = 1.78	0.18	*F*(1,48) = 23.88	<0.01
Attention/executive functions (cs.)	0.06 [−0.20, 0.31]	0.16 [−0.03, 0.36]	0.27 [0.11, 0.42]	*F*(2,48) = 0.66	0.52	*F*(1,48) = 0.07	0.79
ADAS free recall	−0.34 [−0.89, 0.22]	0.15 [−0.36, 0.66]	−0.08 [−0.61, 0.45]	*F*(2,48) = 1.33	0.27	*F*(1,48) = 2.27	0.12
MVGT encoding	0.47 [0.16, 0.78]	0.34 [−0.04, 0.71]	0.66 [0.37, 0.95]	*F*(2,47) = 1.46	0.24	*F*(1,47) = 15.96	< 0.001
MVGT recognition	1.27 [−0.26, 2.80]	0.78 [−0.03, 1.59]	−0.28 [−1.02, 0.46]	*F*(2,46) = 2.35	0.11	*F*(1,46) = 0.14	0.71
Digit span forward	−0.03 [−0.56, 0.50]	−0.19 [−0.71, 0.33]	0.07 [−0.33, 0.47]	*F*(2,48) = 0.58	0.57	*F*(1,48) = 0.23	0.64
Digit span backward	−0.28 [−0.86, 0.30]	0.31 [−0.19, 0.81]	0.14 [−0.29, 0.57]	*F*(2,48) = 0.73	0.40	*F*(1,48) = 0.79	0.38
TMT A	0.36 [0.02, 0.71]	0.22 [−0.14, 0.59]	0.51 [0.12, 0.91]	*F*(2,48) = 0.73	0.49	*F*(1,48) = 2.07	0.16
TMT B	−0.01 [−0.46, 0.43]	0.28 [−0.14, 0.70]	0.20 [−0.03, 0.43]	*F*(2,48) = 0.22	0.81	*F*(1,48) = 1.71	0.20
Phonemic fluency	0.06 [−0.31, 0.43]	0.48 [−0.11, 1.07]	0.45 [0.003, 0.91]	*F*(2,48) = 0.79	0.46	*F*(1,48) = 1.64	0.21
Semantic fluency	0.20 [−0.10, 0.50]	−0.02 [−0.29, 0.26]	0.23 [−1.12, 0.57]	*F*(2,48) = 0.70	0.50	*F*(1,48) = 0.11	0.74
ECB computation span	0.22 [−0.20, 0.65]	0.19 [−0.22, 0.59]	0.33 [−0.03, 0.69]	*F*(2,44) = 0.26	0.77	*F*(1,44) = 2.00	0.16

*Depicted are the mean differences in cognitive measures between pre- and post-training within the three groups and 95% confidence intervals in brackets, as well as statistics for Group × Time and Lifestyle × Time interactions. cs., component score; ADAS, Alzheimer’s Diseases Assessment Scale; MVGT, German adaptation of the California Verbal Learning Test; ECB, Everyday Cognition Battery; TMT, Trail Making Test (part A and B).*

*Values are *z*-standardized on the basis of baseline cognitive assessment.*

### Lifestyle Global Coherence Association and Training Effects on Global Coherence

**FIGURE 2:**
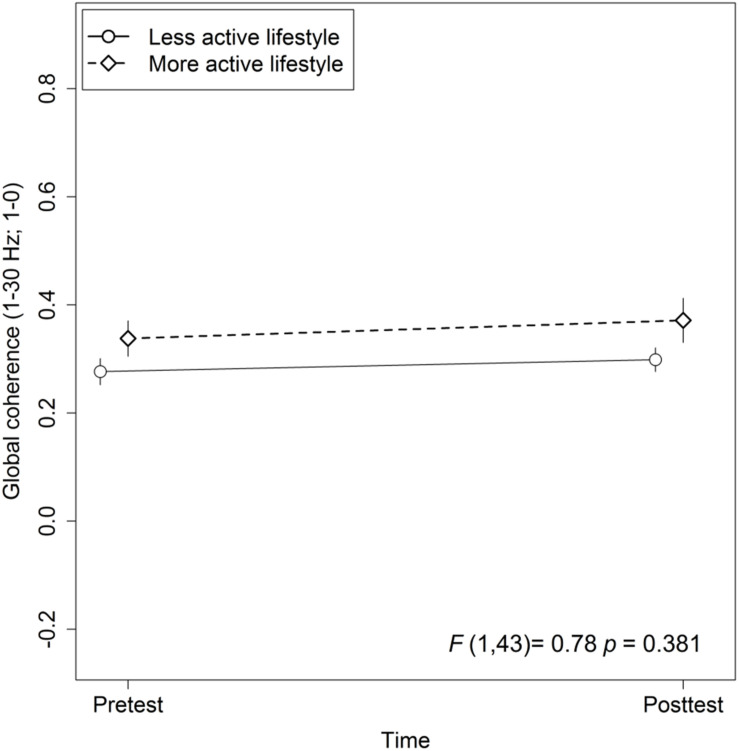
Association between active lifestyle and global coherence. Statistic values refer to the Lifestyle × Time interaction in the Lifestyle × Time + Group × Time linear mixed-effects model. Arrow bars indicate standard errors.

**FIGURE 3:**
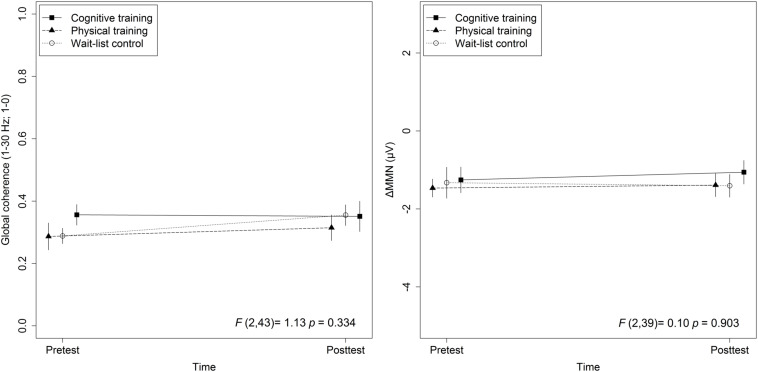
Training effects on global coherence as well as ΔMMN. Statistic values refer to the Group × Time interaction in the Lifestyle × Time + Group × Time linear mixed-effects model. Arrow bars indicate standard errors.

The mixed-effects model comprising Group × Time and Lifestyle × Time as fixed effects and Subject as random intercept with Global coherence as dependent variable revealed no significant associations between lifestyle and global coherence changes over a period of 10 weeks, *F*_(1,43)_ = 0.78, *p* = 0.38 (see Figure 2). The same model revealed no significant training effects on global coherence, *F*_(2,43)_ = 1.13, *p* = 0.33 (see Figure 3). Likewise, in comparison to the control group the Hedges’ *g* was negative for the cognitive, Hedges’ *g* = −0.40, 95% CI [−1.14, 0.33], *p* = 0.29, as well as for the PT, Hedges’ *g* = −0.32, 95% CI [−1.01, 0.37], *p* = 0.34. The secondary analyses did not show a significant change in global coherence over time within groups (see Table 3). The supplemental analysis with ΔMMN calculated from a constant number of 50 trials did no change the main results. The analyses regarding the fronto-parietal and fronto-tempotal area did not reveal significant training-induced effects and lifestyle-associated changes in coherence (see Supplementary Table 2).

**TABLE 3:** Electroencephalography raw data comparisons between pre- and post-training.

	*n*	Pre-training	Post-training	*t* statistic	*p*
**Global coherence**					
Cognitive Training	14	0.36 ± 0.12	0.35 ± 0.18	*t*_(13)_ = 0.08	0.93
Physical Training	17	0.29 ± 0.18	0.31 ± 0.17	*t*_(16)_ = −1.04	0.31
Wait-list control	18	0.29 ± 0.10	0.36 ± 0.14	*t*_(17)_ = −2.21	0.04
**ΔMMN**					
Cognitive Training	10	−1.26 ± 1.05	−1.06 ± 0.96	*t*_(9)_ = −0.45	0.66
Physical Training	16	−1.46 ± 0.92	−1.39 ± 1.19	*t*_(15)_ = −0.34	0.74
Wait-list control	18	−1.33 ± 1.69	−1.41 ± 1.25	*t*_(17)_ = 0.16	0.88

*Values are means (M) ± standard deviations (SD).*

*The *t*-values represent the results of paired samples *t*-tests.*

Δ*MMN, the difference score between MMN for long and for short ISI for duration deviant.*

### Training Effect on ΔMMN

Also, for ΔMMN no significant auditory CT effect was found, *F*_(2,39)_ = 0.10, *p* = 0.90 (see Figure 3). In comparison to the control group the auditory CT did not show beneficial impact on ΔMMN, Hedges’ *g* = 0.14, 95% CI [-0.67, 0.95], *p* = 0.79. The 50 trials’ analyses for ΔMMN did not change the results. The *t*-test as secondary analysis did not reveal a significant pre-post change for ΔMMN (see Table 3).

Also, the *t*-test analyses did not reveal significant alterations in global coherence and ΔMMN within the 10-week period in any of the groups (see Table 3).

### Follow-Up-Assessment

No significant relationships between lifestyle and change in global coherence as well as significant training effects were found after including the follow-up assessment into the model.

## Discussion

This study aimed at investigating the association between an active lifestyle and changes in global coherence in a sample at-risk for developing dementia. Furthermore, we examined the effects of a cognitive and a PT program on global coherence and the effects of auditory CT on ΔMMN-Dur as an index for automatic auditory memory decay.

### Lifestyle Association With Global Coherence

We did not find an association between self-reported lifestyle and changes in global coherence during a period of 10 weeks in older adults at risk for developing dementia. Thus, there is no evidence that global coherence is an underlying mechanism of lifestyle-related cognitive benefits, which were previously reported by several studies including our working group. This finding is not in line with studies reporting positive effects of an enriched environment on neuronal plasticity in animals (e.g., Fabel and Kempermann, 2008; for a review see Kramer et al., 2004), as changes in functional connectivity have been attributed to neuronal structural and functional changes. Notably, the majority of studies investigating the effects of an enriched environment on neuroplasticity reported positive effects for separate areas, while in the present study global changes in functional connectivity were considered. Thus, a possible explanation for the discrepancy in results might be the fact that lifestyle-dependent functional neuroplasticity takes place in individual brain regions or in separate frequency bands and is not reflected by a global coherence score. Finally, the observation period of 10 weeks might have been too short to show significant effects.

### Training Effects on Global Coherence

Contrary to previous results reporting positive training effects on functional connectivity (Voss et al., 2010; Anguera et al., 2013; Klados et al., 2016; Zilidou et al., 2018), we did not find any effects of cognitive or PT interventions on global coherence. So far, only few studies investigated training impacts on functional connectivity. Even if the aforementioned results by previous studies are promising, several methodological differences in investigation tool (functional magnetic resonance imaging, EEG), study sample (healthy adults, cognitively impaired adults), regions of interest (separate regions vs. global connectivity), and training programs make the comparison of study results difficult.

One important factor might be the duration of training. In a functional magnetic resonance imaging study Voss et al. (2010) investigated the effect of aerobic walking training on the default mode network in healthy older adults. Interestingly, the authors reported enhanced coherence in several regions of the default mode network only after a 12-month training period, while the effects were not significant after 6 months. Stronger DMN connectivity was associated with better executive control after training. Consequently, the training period in the present study might have been too short to tap functional neuroplasticity.

In recent years, there is a growing interest in combined training interventions (e.g., Fissler et al., 2013; Herold et al., 2018). Styliadis et al. (2015) found positive effects on MMSE scores only for the combination of an 8-week physical and auditory CT and not for interventions of physical or auditory CT alone. Furthermore, the cognitive improvement was associated with an elevated power in delta and theta band activity which has been shown to be positively associated with neurodegeneration (e.g., Stevens et al., 2001; Adler et al., 2003; Babiloni et al., 2009). Using low resolution brain electromagnetic tomography, Klados et al. (2016) examined the same combined training program used by Styliadis et al. (2015) on EEG coherence and found a widespread training induced elevated beta band coherence. The authors referred training-induced elevation in beta band activity to enhanced neuroplasticity (Styliadis et al., 2015; Klados et al., 2016). Enhanced functional connectivity as a result of neuroplasticity has also been supported by other authors (e.g., Frantzidis et al., 2014; Zilidou et al., 2018). Oswald et al. (2006) offer an explanation for the effectiveness of the combination of physical and auditory CT and conjectured that physical activity drives neuroplasticity induced by cognitively demanding activities through improved metabolic processes. Therefore, the combination of both of the training programs used in the present study might have yielded significant effects, especially because Styliadis et al. (2015) and Klados et al. (2016) used the adapted Greek version of the same auditory CT program that was applied in the present study. Furthermore, similarly to the results for lifestyle, it is also possible that particular brain networks, such as the DMN, and separate frequency bands, such as the beta band, are more sensible to training effects than the global scores (cf., Klados et al., 2016).

### Training Effects on ΔMMN-Dur

In contrast to our hypothesis, we did not find significant auditory CT effects on ΔMMN-Dur. We based our expectation on previous results reporting positive associations between MMN as an index for automatic discrimination ability and the persons’ ability to discriminate changes between sounds or sound sequences. For instance, musicians, who are more capable to discriminate tones in comparison to non-musicians, also showed a larger MMN; their automatically elicited MMN-amplitude was associated with better discrimination performance. Furthermore, an enhanced MMN has been shown to be associated with linguistic skills in young subjects (Tremblay et al., 1997; Kujala et al., 2001; Cheour et al., 2002). It has to be noted that learning a new instrument or a new language might be more intrinsically motivating as exercising an auditory training program. Furthermore, studies on musicians focused on long lasting exercise periods; therefore the comparison to the present 10-week training period is limited. As for the MMN as a marker for linguistic skills, the corresponding studies have been conducted with children or very young adults. Even if the human brain has been shown to stay neuroplastic up to old age (e.g., Gutchess, 2014), neuroplasticity becomes more difficult in advanced age due to age-associated neuronal changes, for example, the neuronal atrophy or hemispheric asymmetry reduction (Oberman and Pascual-Leone, 2013).

While, to our best knowledge, no study exists that investigated the effects of auditory CT on MMN in the field of cognitive aging, more research exists for subjects with schizophrenia or schizophrenia-spectrum illnesses. Persons with schizophrenia-spectrum illnesses show pronounced cognitive deficits. Similar to AD, cognitive deficits in schizophrenia have been shown to be associated with a decreased MMN (e.g., Hermens et al., 2010) and furthermore with functional disability (e.g., Hamilton et al., 2018). Recent studies examined whether auditory training might improve cognition in subjects with schizophrenia and whether the positive changes are accompanied by an improved MMN. In line with the missing training-induced results in the present study, the authors did not find any positive training effects on MMN using the adapted version of the same training as applied in the present study (Kärgel et al., 2016; Biagianti et al., 2017). Some authors even report an attenuated MMN after auditory training in schizophrenia patients (Perez et al., 2017).

These results indicate that some other factors than the auditory training per se might be responsible for the intervention’s success. In this context, Chandrasekaran and Kraus (2010) noted that we learn best about things that we care about, thus the intrinsic motivation, enjoyment, and fun of the training might be of high relevance (cf. also, Küster et al., 2016). These factors might be higher for learning a new instrument or language than for a computer-based relative monotonous training program. Furthermore, the training duration in the aforementioned studies ranged between a single 1-h session (Perez et al., 2017), 2-week training period (Kärgel et al., 2016), 8-week training period (Biagianti et al., 2017), and 10 weeks of training intervention in the present study. Thus, besides the training’s contents and motivational aspects, the missing effect of training in the present study might be explained by the training’s duration.

## Strengths and Limitations

Since global coherence is a global marker measured over the whole skull, it is more reliable as the measure of EEG coherence for separate pairs of electrodes. ΔMMN-Dur as a difference score between MMN for long and for short interstimulus interval is a novel EEG marker which takes different auditory processing aspects into account, such as auditory discrimination ability as well as the automatic auditory memory trace (Näätänen et al., 2012) and is correlated with cognitive performance (Laptinskaya et al., 2018).

Also, the following limitations have to be considered: in our previous research we found a positive lifestyle cognition association but no positive training effects on behavioral measures of cognition. Thus, training-induced electrophysiological changes might be difficult to detect. However, other authors reported positive training effects despite the missing of significant benefits in neuropsychological testing (cf., Miró-Padilla et al., 2020). Random allocation is the best method to prevent selection bias. Due to logistic issues, a randomized allocation to the groups was not feasible. Thus, we chose the minimization approach in order to control for bias regarding age, gender, education, and cognitive status reflected by MMSE. Although, selection bias cannot be excluded, we assume that it was unlikely: there were no differences in sociodemographic data, cognition, or EEG parameters between groups. Finally, the small sample size might be a further limitation.

In the coherence analyses as a measure for functional connectivity, the interactions between brain regions are considered as linear connections, although there are indications that at least some of the interactions between brain regions may have a non-linear component (Rombouts et al., 1995). An often-used non-linear measure for functional connectivity is the synchronization likelihood (SL). In a magnetoencephalography study, Gómez et al. (2009) compared the accuracy of coherence as well as SL to discriminate healthy older adults without cognitive deficits from subjects with MCI. Interestingly, the authors reported a better accuracy for the linear coherence measure. Thus, we assume that coherence analysis is a suitable method to investigate functional connectivity in the present sample at-risk for developing AD.

## Conclusion

In the light of the results of the present study as well as the review of the previous literature, we conclude that training interventions focusing on only one training aspect are not very powerful to generate positive effects on global EEG coherence. Instead, multimodal interventions taking physical and cognitive as well as motivational and emotional aspects into account seem to be more promising. Notably, this sort of intervention is very similar to an active lifestyle, which has been shown to be related to positive cognitive change.

Since, the auditory training did not reveal positive effects on ΔMMN-Dur as an index for automatic auditory memory decay, we suggest that other training parameters such as longer training periods and more intrinsically motivating as well as joyful approaches might be more suitable to tap the automatic auditory memory trace in EEG.

## Data Availability Statement

The raw data supporting the conclusions of this article will be made available by the authors, without undue reservation.

## Ethics Statement

The studies involving human participants were reviewed and approved by the Ulm University and the University of Konstanz. The participants provided their written informed consent to participate in this study.

## Author Contributions

DL contributed to the study conception and design, organized the study procedures and acquired the data, analyzed and interpreted the data, and wrote the first draft of the manuscript. FT contributed to the study conception and design, organized study procedures, acquired the data, contributed to the data analysis, and critically revised the first draft of the manuscript. PF and OK contributed to the study conception and design, organized the study procedures and acquired the data, contributed to the data interpretation and critically revised the manuscript. CA and I-TK conceptualized the study, obtained funding, supervised all phases of the study as principle investigators and critically revised the manuscript. All authors read and approved the final manuscript.

## Conflict of Interest

The authors declare that the research was conducted in the absence of any commercial or financial relationships that could be construed as a potential conflict of interest.

## Abbreviations

AD: Alzheimer’s disease
ADAS: Alzheimer’s Disease Assessment Scale
ANOVA: analysis of variance
CT: auditory cognitive training
DMN: default mode network
EEG: electroencephalography
fMRI: functional magnetic resonance imaging
ISI: interstimulus interval
MCI: mild cognitive impairment
MMN: mismatch negativity
MMSE: Mini-Mental State Examination
MVGT: Münchner Verbaler Gedächtnistest
PT: physical training
SL: synchronization likelihood
SOA: stimulus onset asynchrony
WLC: wait-list control
Δ MMN: difference score between MMNs to short and to long ISIs
Δ MMN–Dur: index for automatic auditory memory amplitude difference between the MMN after duration deviant in the Memory Trace paradigm and Optimum–1 paradigm higher values indicating better automatic auditory memory or less pronounced automatic auditory memory decay.
