# Supplementary material for: No Evidence That Cognitive and Physical Activities Are Related to Changes in EEG Markers of Cognition in Older Adults at Risk of Dementia
Source: Front Aging Neurosci. 2021 Mar 19;13:610839. doi: 10.3389/fnagi.2021.610839 (PMC8017171; doi:10.3389/fnagi.2021.610839)
Supplement: Supplementary file 1 [file Presentation_1.pdf]

Supplementary Material

No evidence that cognitive and physical activities are related to changes in EEG markers of cognition in older adults at risk of dementia

Daria Laptinskaya[1,2*], Olivia Caroline Küster[3,4], Patrick Fissler[1,3,5], Franka Thurm[6,2], Christine A. F. von Arnim[3,7], & Iris-Tatjana Kolassa[1,2]

*** Correspondence:** Daria Laptinskaya: daria.laptinskaya@uni-ulm.de, daria.laptinskaya@gmail.com
Iris-Tatjana Kolassa: iris.kolassa@uni-ulm.de

Classification of Cognitive Groups

Participants' cognitive status was classified into four categories: subjective memory impairment (SMI), amnestic mild cognitive impairment (aMCI), non-amnestic mild cognitive impairment (naMCI), and probable dementia. All participants reported subjective cognitive impairment, which was assessed with the question "Do you feel like your memory is getting worse?" (according to Geerlings et al., 1999 and Jessen et al., 2010). The evaluation of objective cognitive impairment was based on encoding (sum of words of the five learning trials) and long-delay free recall scores of the adapted German version of the California Verbal Learning Test (German: Münchner Verbaler Gedächtnistest [MVGT, Munich Vebal Memory Test]; Ilmberger, 1988) for memory functions. For non-memory cognitive functions the following subtests from the Consortium to Establish a Registry for Alzheimer's Disease–plus (Welsh et al., 1994) were used: Trail Making Test (TMT) part A and B, phonematic and semantic word fluency, and Boston Naming Test. Objective cognitive impairment was defined as 1.0 *SD* below the age- and education-adjusted norm. Participants with subjective, but no objective impairment were classified as SCI. A participant was classified as aMCI, if at least one of the memory tests was below average. naMCI was assigned, if performance in the memory tests was average while one of the test scores of the other cognitive domains was below average. Severe objective impairment (≤ 2 *SD* below the norm) in memory and non-memory indicated probable dementia. In four of five cases an experienced neurologist or psychiatrist confirmed the diagnosis of probable dementia.

MMN Analysis

The following number of trials was left for averaging in the Optimum–1 paradigm (values are means ± standard deviations): 750 ± 54 for the standard tone as well as 151 ± 12 for the duration deviant in the pre-assessment; 728 ± 17 as well as 147 ± 17 trials in the post-assessment; and 740 ± 69 as well as 147 ± 14 trials in the follow-up-assessment, respectively. In the Memory Trace paradigm, the electrophysiological signal after the standard tone was averaged over 83 ± 5 trials and after the duration deviant over 66 ± 4 trials in the pre-assessment; over 81 ± 9 after standard tone and over 61 ± 8 after duration deviant in the post-assessment; and over 87 ± 8 as well as 67 ± 6 in the follow-up-assessment, respectively. Finally, the data were re-referenced to the linked mastoids.

Supplementary ΔMMN Analyses

To avoid confounding effects of the trial number of the averaged trials, main analyses were repeated by building the ΔMMN from 50 randomly selected artifact-free trials for each assessment and each subject. The trial number of 50 has been shown to provide reliable results in clinical samples (cf., Marco-Pallares et al., 2011). In the Memory Trace paradigm, for a few subjects (two in the pre-assessment, four in the post-assessment, and one in the follow-up-assessment), only a smaller number of trials (43-49, and 31 trials for one subject in one assessment) was available. For these participants the analyses were repeated with the available number of trials.

References

Geerlings, M. I., Jonker, C., Bouter, L. M., Adèr, H. J., and Schmand, B. (1999). Association between memory complaints and incident Alzheimer's disease in elderly people with normal baseline cognition. *Am. J. Psychiatry* 156, 531–537. doi:10.1176/ajp.156.4.531

Ilmberger, J. (1988). *Münchner Verbaler Gedächtnistest (MVGT) [Munich Verbal Memory Test, unpublished manuscript]*. München: Institut für Medizinische Psychologie, Universität München.

Jessen, F., Wiese, B., Bachmann, C., Eifflaender-Gorfer, S., Haller, F., Kölsch, H., et al. (2010). Prediction of dementia by subjective memory impairment: effects of severity and temporal association with cognitive impairment. *Arch. Gen. Psychiatry* 67, 414–422. doi:10.1001/archgenpsychiatry.2010.30

Marco-Pallares, J., Cucurell, D., Münte, T. F., Strien, N., and Rodriguez-Fornells, A. (2011). On the number of trials needed for a stable feedback-related negativity. *Psychophysiology*. doi:10.1111/j.1469-8986.2010.01152.x

Welsh, K. A., Butters, N., Mohs, R. C., Beekly, D., Edland, S., Fillenbaum, G., et al. (1994). The Consortium to Establish a Registry for Alzheimer's Disease (CERAD). Part V. A normative study of the neuropsychological battery. *Neurology* 44, 609–614. doi:10.1212/WNL.44.4.609
