# Supplementary material for: No Evidence That Cognitive and Physical Activities Are Related to Changes in EEG Markers of Cognition in Older Adults at Risk of Dementia
Source: Front Aging Neurosci. 2021 Mar 19;13:610839. doi: 10.3389/fnagi.2021.610839 (PMC8017171; doi:10.3389/fnagi.2021.610839)
Supplement: Supplementary file 2 [file Table_1.docx]

Supplementary Material

**No evidence that cognitive and physical activities are related to changes in EEG markers of cognition in older adults at risk of dementia**

Daria Laptinskaya^1,2*^, Olivia Caroline Küster^3,4^, Patrick Fissler^1,3,5^, Franka Thurm^6,2^, Christine A. F. von Arnim^3,7^, & Iris-Tatjana Kolassa^1,2^

*** Correspondence:** Daria Laptinskaya: [daria.laptinskaya@uni-ulm.de](mailto:daria.laptinskaya@uni-ulm.de), [daria.laptinskaya@gmail.com](mailto:daria.laptinskaya@gmail.com) Iris-Tatjana Kolassa: iris.kolassa@uni-ulm.de

| **Supplementary Table 1. Categorization of activities into social, physical, and cognitive domains (cf., Küster et al., 2016, additional methods, Table A1).** | | | | | |
| --- | --- | --- | --- | --- | --- |
|  | **Rating scores** | | |  |  |
|  | **Cognitive domain** | **Social**  **domain** | **Physical domain** |  | **%Subjects** |
| **Multidomain activities^a^** |  |  |  |  |  |
| Play basketball, soccer or racquetball | 3.7 | 4.3 | 5.0 |  | 10 |
| Play singles tennis | 3.3 | 3.7^d^ | 5.0 |  | 6 |
| Play doubles tennis | 3.3 | 3.3 | 5.0 |  | 0 |
| Dance | 3.3 | 4.3 | 4.0 |  | 13 |
| Play cards and board games | 4.3 | 4.7 |  |  | 29 |
| Visit family or friends | 3.3 | 5.0 |  |  | 81 |
| Do volunteer work | 4.0 | 4.0 |  |  | 56 |
| Attend club meetings | 3.3 | 4.7 |  |  | 50 |
| Attend cultural events | 4.0 | 3.3 |  |  | 62 |
| Do Yoga or Tai Chi | 3.5^d^ |  | 3.3 |  | 4 |
| Do aerobic |  | 3.5^d^ | 4.7 |  | 0 |
| Play golf, with carrying equipment |  | 3.6^d^ | 3.8^d^ |  | 0 |
| **Single domain activities^b^** |  |  |  |  |  |
| Play musical instruments | 5.0 |  |  |  | 10 |
| Use a computer | 4.3 |  |  |  | 73 |
| Read | 3.7 |  |  |  | 98 |
| Do arts and crafts | 3.3 |  |  |  | 38 |
| Go to the senior center |  | 4.3 |  |  | 25 |
| Attend church activities |  | 3.3 |  |  | 38 |
| Jog or run |  |  | 4.7 |  | 21 |
| Swim moderately or fast |  |  | 4.7 |  | 13 |
| Skate (ice, roller, in-line) |  |  | 4.0 |  | 2 |
| Use an aerobic machine |  |  | 4.3 |  | 19 |
| Do moderate/heavy strength training |  |  | 4.3 |  | 15 |
| Walk uphill or hike |  |  | 3.7 |  | 46 |
| Do heavy gardening |  |  | 3.7 |  | 35 |
| Do water exercise |  |  | 3.7 |  | 19 |
| Bicycle |  |  | 3.7 |  | 71 |
| Do heavy work around the house |  |  | 3.3 |  | 50 |
| **Low demand activities^c^** |  |  |  |  |  |
| Play golf, riding in a cart |  |  |  |  | 0 |
| Shot pool or billiards |  |  |  |  | 2 |
| Do light work around the house |  |  |  |  | 88 |
| Do light gardening |  |  |  |  | 50 |
| Work on machinery |  |  |  |  | 23 |
| Walk fast or briskly |  |  |  |  | 33 |
| Walk to do errands |  |  |  |  | 71 |
| Walk leisurely |  |  |  |  | 62 |
| Swim gently |  |  |  |  | 21 |
| Do stretching or flexibility |  |  |  |  | 58 |
| Do light strength training |  |  |  |  | 23 |
| General conditioning exercises |  |  |  |  | 25 |
| *Depicted are mean ratings on a five-point rating scale from 1 (no demands) to 5 (high demands), for ratings higher than 3. % Subjects = Percentage of subjects who had engaged into the respective activity. ^a^Two and three domains with rating > 3. ^b^One domain with rating > 3. ^c^No domain with rating > 3. ^d^Categorization adapted to senior ratings.* | | | | | |

**Reference**

Küster, O. C., Fissler, P., Laptinskaya, D., Thurm, F., Scharpf, A., Woll, A., et al. (2016). Cognitive change is more positively associated with an active lifestyle than with training interventions in older adults at risk of dementia: a controlled interventional clinical trial. *BMC Psychiatry* 16, 315. doi:10.1186/s12888-016-1018-z.
