# Supplementary material for: No Evidence That Cognitive and Physical Activities Are Related to Changes in EEG Markers of Cognition in Older Adults at Risk of Dementia
Source: Front Aging Neurosci. 2021 Mar 19;13:610839. doi: 10.3389/fnagi.2021.610839 (PMC8017171; doi:10.3389/fnagi.2021.610839)
Supplement: Supplementary file 3 [file Table_2.docx]

Supplementary Material

**No evidence that cognitive and physical activities are related to changes in EEG markers of cognition in older adults at risk of dementia**

Daria Laptinskaya^1,2*^, Olivia Caroline Küster^3,4^, Patrick Fissler^1,3,5^, Franka Thurm^6,2^, Christine A. F. von Arnim^3,7^, & Iris-Tatjana Kolassa^1,2^

**Correspondence:** Daria Laptinskaya: [daria.laptinskaya@uni-ulm.de](mailto:daria.laptinskaya@uni-ulm.de), [daria.laptinskaya@gmail.com](mailto:daria.laptinskaya@gmail.com)

Iris-Tatjana Kolassa: iris.kolassa@uni-ulm.de

| **Supplementary Table 2. Training and lifestyle-related changes in cognition from pre- to post-training.** | | | | | | | | | | | | | | | | |  |
| --- | --- | --- | --- | --- | --- | --- | --- | --- | --- | --- | --- | --- | --- | --- | --- | --- | --- |
|  |  | **Difference post-pre [95% CI]** | | | | |  | **Group × Time** | | |  | | **Lifestyle × Time** | | | | |
| **Outcome**  **coherence measure** |  | **Cognitive training (*n* = 14)** |  | **Physical training (*n* = 17)** |  | **Wait-list control (*n* = 18)** |  | ***F* statistic** |  | ***p*** | |  | | ***F* statistic** |  | ***p*** | |
| **Fronto-temporal** |  | 0.03 [0.01., 0.06] |  | 0.04 [0.01, 0.07] |  | 0.03 [0.01, 0.05] |  | *F*_(2,43)_ = 0.59 |  | 0.56 | |  | | *F*_(1,43)_ = 0.14 |  | 0.72 | |
| **Fronto-pariental** |  | 0.02 [-0.004, 0.04] |  | 0.04 [0.01, 0.07] |  | 0.04 [0.01, 0.06] |  | *F*_(2,43)_ = 1.79 |  | 0.18 | |  | | *F*_(1,43)_ = 0.49 |  | 0.49 | |
| *Depicted are the mean differences in global coherence (1-30 Hz) measure for the fronto-temporal and the fronto-temporal region within the three groups and 95% confidence intervals in brackets, as well as statistics for Group × Time and Lifestyle × Time interactions.* | | | | | | | | | | | | | | | | |  |
